# The cost of sympatry: spatio-temporal patterns in leopard dietary and physiological responses to tiger competition gradient in Rajaji Tiger Reserve, Uttarakhand, India

**DOI:** 10.1093/conphys/coad039

**Published:** 2023-05-29

**Authors:** Shiv Kumari Patel, Sourabh Ruhela, Suvankar Biswas, Supriya Bhatt, Bivash Pandav, Samrat Mondol

**Affiliations:** Wildlife Institute of India, Chandrabani, Dehradun, Uttarakhand 248001, India; Wildlife Institute of India, Chandrabani, Dehradun, Uttarakhand 248001, India; Wildlife Institute of India, Chandrabani, Dehradun, Uttarakhand 248001, India; Wildlife Institute of India, Chandrabani, Dehradun, Uttarakhand 248001, India; Wildlife Institute of India, Chandrabani, Dehradun, Uttarakhand 248001, India; Wildlife Institute of India, Chandrabani, Dehradun, Uttarakhand 248001, India

**Keywords:** stress and nutrition hormones, physiological cost, inter-species competition, habitat recovery, dominance hierarchy, Carnivore conservation

## Abstract

Apex predators have critical roles in maintaining the structure of ecosystem functioning by controlling intraguild subordinate populations. Such dominant–subordinate interactions involve agonistic interactions including direct or indirect impacts on the subordinates. As these indirect effects are often mediated through physiological processes, it is important to quantify such responses to better understand population parameters. We used a large carnivore intraguild system involving tiger (*Panthera tigris*) and leopard (*Panthera pardus*) to understand the dietary and physiological responses under a spatio-temporal gradient of tiger competition pressures in Rajaji Tiger Reserve (RTR) between 2015 and 2020. We conducted systematic faecal sampling in the winters of 2015 and 2020 from the park to assess diet and physiological measures. Analyses of leopard-confirmed faeces suggest a dietary-niche separation as a consequence of tiger competition. In 2020, we found an increased occurrence of large-bodied prey species without tiger competition in western-RTR. Physiological measures followed the dietary responses where leopards with large-sized prey in the diet showed higher fT3M and lower fGCM measures in western-RTR. In contrast, eastern-RTR leopards showed lower levels of fT3M and fGCM in 2020, possibly due to intense competition from tigers. Overall, these patterns strongly indicate a physiological cost of sympatry where competition with dominant tigers resulted in elevated nutritional stress. We recommend expansion of leopard monitoring and population estimation efforts to buffers, developing appropriate plans for human–leopard conflict mitigation and intensive efforts to understand leopard population dynamics patterns to ensure their persistence during the ongoing Anthropocene.

## Teaser

Leopards showed spatio-temporal dietary and physiological responses againts tiger competition in Rajaji Tiger Reserve, India. Large-sized prey in the leopard diet correlated with better physiological status without competition. Areas with intense tiger competition resulted in poorer physiological measures. Patterns strongly indicate a physiological cost of sympatry within the tiger-leopard guild.

## Introduction

Apex predators are critical in maintaining the structure and control of the local ecosystem functioning through prey–predator dynamics (Ritchie and Johnson, 2009; Ritchie *et al.,* 2012; Ripple *et al.,* 2014; Gaynor *et al.,* 2019) and their limiting effects on subordinates (Ritchie and Johnson, 2009; Ripple *et al.,* 2014; Suraci *et al.,* 2016; Feit *et al.,* 2019). Intensity of such limiting effects within a large carnivore guild is more pronounced for species competing for similar resources (Palomares and Caro, 1999; Donadio and Buskirk, 2006; Ritchie and Johnson, 2009). Dominant species within the guild exercise interference competition through aggression (Linnell and Strand, 2000; Merkle *et al.,* 2009), harassment (Merkle *et al.,* 2009), kleptoparasitism (Merkle *et al.,* 2009; Périquet *et al.,* 2015), delaying intraspecific communication (Cornhill and Kerley, 2020) and direct killing (Laurenson, 1994; Palomares and Caro, 1999; Donadio and Buskirk, 2006; Merkle *et al.,* 2009; Ritchie and Johnson, 2009). Subordinate members, on the other hand employ various tactics (for example, spatio-temporal and dietary separation) to minimize interference and maximize resource acquisition (Hayward and Slotow, 2009; Vanak *et al.,* 2013) to achieve a balance for successful co-existence. Local ecological factors are known to drive such behavioral strategies, which has been an area of extensive research interest in various carnivore guilds globally (Karanth and Sunquistt, 1995; Karanth and Sunquist, 2000; Carlsson *et al.,* 2010; Broekhuis *et al.,* 2013; Steinmetz *et al.,* 2013; Vanak *et al.,* 2013; Périquet *et al.,* 2014; López-Bao *et al.,* 2016). Dominant–subordinate agonistic interactions exerts two different kinds of negative impacts on the subordinate species: (a) direct impacts such as getting killed or displacement from the best-quality habitats (Mitchell and Banks, 2005; Merkle *et al.,* 2009; Newsome *et al.,* 2017; Ramesh *et al.,* 2017) and (b) indirect effects from increased pressures from competitions, inadequate food resources and resulting energy deficits (Creel and Christianson, 2008; Suraci *et al.,* 2016; Creel *et al.,* 2017; Sheriff *et al.,* 2020) affecting survival, growth, body condition, reproduction and parental provisioning (Creel *et al.,* 2007, 2009; Creel and Christianson, 2008; Parker *et al.,* 2009; Lamanna and Martin, 2016). As many of these indirect effects are mediated through physiological processes (Clinchy *et al.,* 2013; Macleod *et al.,* 2018), quantification of the physiological responses is essential to understand changes in various population parameters of the subordinate species (Creel *et al.,* 2009; Gaynor *et al.,* 2019). Recent advances in physiological measurements of environmental stressors, particularly in combination with non-invasive sampling approaches, have made it easier to link the environmental effects with their respective physiological responses (Dantzer *et al.,* 2014; Sopinka *et al.,* 2015; Palme, 2019; Ames *et al.,* 2020). For example, a number of inter-species (predator–prey—see Ylönen *et al.,* 2006; Creel *et al.,* 2009, dominant–subordinate dynamics-see Van Meter *et al.,* 2009 etc.) and intra-species (social hierarchy-see Sapolsky, 1983; Armitage, 1991; Creel *et al.,* 1996; Creel, 2001; Van Meter *et al.,* 2009, competition-see Armitage, 1991; Creel, 2001) interactions have been addressed using glucocorticoid (GC) measures, demonstrating its use. Further, recent addition of thyroid hormone (T3, in particular) (Eales, 1988; Flier *et al.,* 2000; Wasser *et al.,* 2010; Behringer *et al.,* 2018) measure is allowing us to separate the impacts of dietary resource availability from overall stress measures (through GC) as shown in marine (Ayres *et al.,* 2012; Wasser *et al.,* 2017; McCormley *et al.,* 2018) and terrestrial mammals (Wasser *et al.,* 2011; Vynne *et al.,* 2014; Joly *et al.,* 2015; Dias *et al.,* 2017; Szott *et al.,* 2020), including large carnivores (Vynne *et al.,* 2014; Patel *et al.,* 2021).

**Figure 1:**
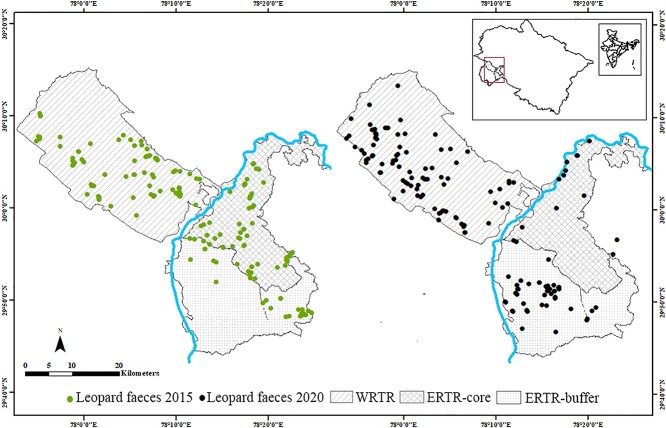
Spatio-temporal locations of field-collected leopard faecal samples from Rajaji Tiger Reserve (RTR) used in this study. The core and buffer zones are differentially marked. The left pane represents samples collected in 2015, whereas the right pane shows 2020 samples.

The sympatric tiger (*Panthera tigris*) and leopard (*Panthera pardus*) are one of the most well-studied model systems to understand the dominant–subordinate intraguild competition (Seidensticker, 1976; McDougal, 1988; Mondal *et al.,* 2012; Steinmetz *et al.,* 2013; Carter *et al.,* 2015; Pokheral and Wegge, 2019; Kafley *et al.,* 2019; Kumar *et al.,* 2019; Thapa *et al.,* 2021). Leopards, when co-existing with tigers, are often dominated by their larger counterpart in terms of resources (both space and food) (Seidensticker, 1976). Large number of studies have focused on exploring different strategies adopted by leopards, such as spatio-temporal (Carter *et al.,* 2015; Kafley *et al.,* 2019; Kumar *et al.,* 2019; Pokheral and Wegge, 2019; Thapa *et al.,* 2021) and dietary niche segregation (Karanth and Sunquistt, 1995; Andheria *et al.,* 2007; Harihar *et al.,* 2011; Pokheral and Wegge, 2019) for successful co-existence with tigers, but the physiological consequences of such interactions have received less attention. Here we address leopard physiological and dietary responses in the context of competition with tigers within Rajaji Tiger Reserve (RTR), western Terai-Arc landscape, India. RTR is a major source tiger population (estimated density of 8 ± 1.4/100 km^2^ in 2018) and retains one of the highest density of leopards (16.90 ± 1.44/100km^2^) in the landscape (Jhala *et al.,* 2021). The park is physically separated by the river Ganges in two parts: eastern and western RTR (henceforth ERTR and WRTR) that are structurally connected by a narrow corridor, heavily affected by anthropogenic activities (Johnsingh *et al.,* 1990; Harihar *et al.,* 2018; Biswas *et al.,* 2022a) (Figure 1). Both sites are similar in terms of wild prey densities and vegetation structure (Harihar *et al.,* 2009b) but differ in the extent of tiger competition intensity. Almost the entire tiger population of RTR is found in the ERTR whereas leopard, in the absence of tiger, is functionally the apex predator in the WRTR. This unique situation provides an ideal, natural system to assess the physiological impacts of inter-species competition in a control-test framework (WRTR is a control site with no inter-species competition when compared with ERTR). We used leopard faecal hormone metabolite measurements (fGCM and fT3M) in 2015 and 2020 to address spatio-temporal differences in physiological and dietary responses against a tiger competition gradient. More specifically, we asked the following questions: (i) how leopard dietary profiles, fGCM and fT3M measures vary with changing tiger competition intensities over space and time, and (ii) how local ecological factors (habitat productivity and prey body size) explain such differences in leopard physiology. We believe that results of this study have larger implications in understanding the physiological costs for subordinate carnivores co-existing within a guild and their long-term conservation.

## Materials and Methods

### Research permissions and ethical considerations

All required permissions for field surveys and faecal sampling were provided by Forest Departments of Uttarakhand (permit no: 90/5-6 and permit no: 3707/5-6). Due to the non-invasive nature of this study, no further ethical clearance was required.

### Study area

This study was conducted in Rajaji Tiger Reserve (RTR) (Figure 1), the westernmost part of Rajaji-Corbett Tiger Conservation Unit (RCTCU, Johnsingh and Negi, 2003), in the Indian part of the Terai-Arc landscape (TAL). Located at base of Himalayan foothills and starting of Indo-Gangetic plains, RTR has an undulating topography with a mosaic of woodlands and grasslands, drained by multiple rivers and streams. The forest type is broadly classified as northern Indian moist deciduous, dominated by Sal (*Shorea robusta*) (Champion and Seth, 2005). The park is naturally separated by the river Ganges in two parts, connected by a narrow corridor called as Chilla-Motichur corridor (3 km length and 1 km width) (Johnsingh *et al.,* 1990; Harihar *et al.,* 2018). The eastern part (covering 579 km^2^ area of core and buffer zones), situated in the east bank of Ganges maintains structural, as well as functional connectivity with major tiger populations (such as Corbett Tiger Reserve) of the landscape (Harihar *et al.,* 2020; Biswas *et al.,* 2022a). However, the western part (571 km^2^ area on the west bank of Ganges) has become isolated over years from the ERTR. Historically, the entire study area (RTR) was inhabited by large numbers of agro-pastoralist community settlements (Toungya and Gujjars, respectively), which were primarily dependent on forest resources (Berkmuller *et al.,* 1987), leading to forest degradation. In 1983, Rajaji was declared as a national park and significant efforts towards tiger and prey population recovery were undertaken. As part of this, a plan for relocation and rehabilitation of local communities was prepared to create inviolate space for wildlife (Roy, 2003), and accordingly, all settlements from the core areas of ERTR were relocated by 2003 (Harihar *et al.,* 2009b). Subsequently, the conditions of the natural habitat improved and the densities of the wild prey species increased and facilitated population recovery of wild tigers in ERTR (from 2.08 in 2004–5 to 7.05 individuals/100 km^2^ in 2016–17, Harihar *et al.,* 2020). However, in the WRTR, the human rehabilitation was completed in periodical manner where by 2005 some ranges were made inviolate (Harihar *et al.,* 2009b) and others were completed by 2016. As WRTR remained functionally disconnected from eastern counterpart during this period, the tiger population reduced drastically (from 5 to 10 individuals in 2000 to two females in 2006, Johnsingh, 2006, Jhala *et al.,* 2020) despite comparable prey density (41.22 ± 6.65 individuals/km^2^ in WRTR and 39.23 ± 4.76 individuals/km^2^ in ERTR, Harihar *et al.,* 2020). The leopard population, on the other hand, showed a reverse trend where ERTR recorded a decrease in their density (9.76 to 2.07 individuals/100 km^2^ between 2004 and 2008, respectively, Harihar *et al.,* 2011) (possibly due to inter-species competition) and WRTR showed an increasing trend in leopard density (Jhala *et al.,* 2021) (due to less competition from tigers). Such contrasting population patterns under a scenario of inter-species competition provided an ideal ‘natural experimental setup’ to understand leopard physiological responses under low and high tiger density in adjacent and similar habitats. Further, we conducted this study over a 5-year temporal framework (2015 and 2020) during which the ERTR has seen a significant increase in tiger population (2.90 ± 0.87 to 8 ± 1.4/100 km^2^, Jhala *et al.,* 2015 and 2020), therefore providing an opportunity to test the effects of competition against a tiger density gradient (high tiger density in 2020 than 2015 within ERTR).

### Study design

The unique situation of naturally high (ERTR) vs. low (WRTR) tiger density and reverse patterns of leopard density in adjacent and similar habitats allowed us to ask some important questions regarding various effects of inter-species competition. From a spatial difference perspective we hypothesize that: (1) there would be a dietary niche separation between ERTR and WRTR, where the eastern leopard population would show higher frequency of medium and small sized prey in their diet than the western population (where they do not face competition from tigers); (2) corresponding high fGCM in ERTR (due to inter-species competition) than western population; and (3) lower fT3M in ERTR leopards (due to possible dietary niche separation) than their western counterpart. In a temporal data perspective, we expect that (4) there would be no significant difference in prey relative frequency of occurrence (RFO) in diet, as well as in fGCM and fT3M measures in WRTR, whereas (5) significant difference in prey RFO in diet, fGCM and fT3M is expected in ERTR resulting from increased competition from tigers between 2015 and 2020 (tiger density of 2.90 ± 0.87/100 km^2^ in 2015 and 8 ± 1.4/100 km^2^ in 2020, Jhala *et al.,* 2020). Apart from the inter-species competition we also expected habitat productivity-related differences in leopard dietary and physiological responses. We expected better habitat to be associated with higher frequency of large sized prey in diet, lower fGCM and higher fT3M levels.

### Faecal sampling, species confirmation and prey identification

Due to the spatio-temporal nature of the study, it was critical to establish a standard sampling framework for faecal collection from the entire study area. Some of the major concerns were identification of sampling trails across RTR, seasonal effects, uniform field efforts, constant sampling team etc. As RTR has been a long-term study site for photographic monitoring of tigers (Harihar *et al.,* 2009a, 2009b, 2020; Harihar and Pandav, 2012) and earlier genetic studies have used already identified forest trails and tracks (Biswas *et al.,* 2022a), the information was used for faeces collection in this study during both sampling periods (2015 and 2020). Two experienced research and tracking team (each consisting of three to four members) surveyed all identified forest trails during 2015 and 2020 and collected fresh faecal samples of large carnivores. Both sampling (in 2015 and 2020) were conducted in winter (December–January) to counter seasonal variations and make use of better environmental conditions (mean temperature of 4–6°C) that maintain relatively long-term sample freshness. In field, sample freshness was determined based on intactness, minimal insect activity and strong odor (Vynne *et al.,* 2014). All fresh faecal samples were collected in wax paper with location details and stored in zip-lock bags (Biswas *et al.,* 2019) before transporting them to the laboratory, where they were stored −20°C until laboratory analysis.

In the laboratory, the samples were genetically ascertained using leopard-specific molecular markers (Mondol *et al.,* 2014) to ensure only confirmed leopard samples were used in downstream diet and physiological analyses. In brief, DNA extraction was performed using a modified Qiagen DNA extraction protocol (Biswas *et al.,* 2019) for all samples and leopard-specific mitochondrial DNA markers (TigParND4-F and ParND4-R, Mondol *et al.,* 2014) were used to ascertain leopard faeces. Confirmed leopard samples were further dried at 60°C for 72 hours in an oven (Unilab-112HO, Haryana, India) to control for moisture (Wasser *et al.,* 1993). The undigested parts (prey hair, broken bones, hoof etc.) were separated by sieving the dried samples through sterile 0.5 mm stainless steel mesh and the faecal powders were collected and stored in −20°C. The primary guard hairs (20–30 hairs/sample) were used to prepare permanent slides and were examined for medulla structures (Mukherjee *et al.,* 1994) using available references (Bahuguna *et al.,* 2010; Biswas *et al.,* 2022b) to identify leopard prey species. Sample size estimation for diet analyses was conducted through a sample rarefaction curve (Magurran, 2004), where the species diversity in leopard diet was estimated using Shannon diversity index (Magurran, 2004) with EstimateS (Colwell, 2006).

### Habitat productivity assessment

For leopards or for large carnivores in general a good quality habitat is one with good prey availability (Carbone and Gittleman, 2002). Prey availability is associated with forage availability that is often quantified in terms of vegetation cover or green cover (Pettorelli *et al.,* 2005a, 2005b, 2011). We used vegetation cover as a proxy of habitat productivity (Pettorelli *et al.,* 2011) that would facilitate higher prey base for leopards and quantified it by extracting Normalized Difference Vegetation Index (NDVI) values. We used 16-day composite NDVI values recorded by NASA’s MODIS (Moderate Resolution Imaging Spectroradiometer, MOD13Q1 version 6.1 at 250 m resolution), downloaded for RTR (for the month of December, corresponding to winter sampling season) for year 2015 and 2020. The analyses were conducted at two scales: (a) For overall habitat productivity assessment, we divided study area into three zones: WRTR, ERTR-core and ERTR-buffer (see Fig. 1). Each zone was further divided into 3 × 3 km grids (9 km^2^ area, approximate leopard home range see Seidensticker *et al.,* 1990); and (b) for sample-based assessment, we used leopard faecal sample location as center and drew buffers of 2 km radius (12 km^2^ area) around each faecal sample. We extracted mean NDVI values for each grid and buffer using MODIS raster images of year 2015 and 2020, where extraction was done using zonal statistics tool (as table for grids and table 2 for buffers) in ArcMap 10.5 (ESRI 2016).

### Hormone metabolite extraction and assays

Recent study on wild tigers in the same landscape showed highly variable contents of inorganic matter (IOM) in the faeces that negatively impacted fGCM and fT3M measures (Patel *et al.,* 2021). As leopards share the same space, environmental conditions and prey base in RTR, the field-collected samples were processed for percent IOM measures using the same approach described in Patel *et al.* (2021). In brief, 0.1 g of faecal powder was ashed in a muffle furnace (NSW-101, NSW, New-Delhi, India) at 550°C for 2 hours, reweighed and the amount of IOM was calculated. As suggested in the earlier study (Patel *et al.,* 2021), leopard samples with < 80% IOM content were used for hormone assays.

For hormone metabolite extractions, each dried faecal powder was thoroughly mixed and 0.1 g of powder was weighed. The extraction procedure involved pulse-vortexing the weighed amount of faecal powder in 15 ml of 70% ethanol for 30 minutes, followed by centrifugation at 2200 rpm for 20 min (Wasser *et al.,* 2010; Mondol *et al.,* 2020). The hormone extracts were collected in 2 ml cryochill vials (1:15 dilution) and stored at −20°C in freezer until assays. Leopard fGCM and fT3M were measured using corticosterone (K014, Arbor Assays, MI, USA) and triiodothyronine (T3) (K056, Arbor Assays, MI, USA) EIA kits. These kits were earlier successfully validated in wild tigers (from TAL) and captive lions (Goswami *et al.,* 2021; Patel *et al.,* 2021) and thus were considered suitable for this study. Further, parallelism and accuracy tests were conducted for leopard faecal extracts in the laboratory. Serial dilutions of faecal extracts paralleled well with standard curves of fGCM (Supplementary Material, Fig. S1a), as well as fT3M (Supplementary Material, Fig. S1c). *F* ratio test showed no differences between slopes of standard and pooled extract curves for fGCM (*F*(1,10) = 1.89, *P* = 0.2) and fT3M (*F*(1,11) = 1.34, *P* = 0.27). Accuracy tests using regression analysis produced slopes of 1.09 and 1.02 at working dilution of 1:120 and 1:7.5 for fGCM and fT3M (Supplementary Material, Fig. S1b and S1d). Intra-assay coefficient of variation (CV) was 7.15 and 8.36, whereas inter-assay CV was 10.35 and 7.86 for fGCM and fT3M, respectively. During assays, hormone extracts were dried and reconstituted in assay buffers at required dilution (1:120 for fGCM and 1:7.5 for fT3M). Samples were assayed in duplicate using kit protocols and optical density (at 450 nm) was measured with ELISA plate reader (GMB-580; Genetix Biotech Asia, New Delhi, India). Cross-reactivities of respective antibodies are presented in Supplementary Material, Table S1.

### Statistical analysis

The analytical framework was established based on the hypothesis proposed in this study, where comparisons were made at two scales: 1) at spatial level, prey and hormone metabolite (fGCM and fT3M) data between ERTR and WRTR (individually the 2015 and 2020 data); and 2) at temporal scale where comparisons were made with each part of RTR (2015 vs 2020 for ERTR and WRTR, respectively). While reporting the methods and results following terms have been used to describe the sampled groups: ERTR in 2015—ERTR_2015_, ERTR in 2020—ERTR_2020_, WRTR in 2015—WRTR_2015_ and WRTR in 2020—WRTR_2020_.

To ascertain leopard food habit, data on relative frequencies of occurrences (RFO) for each prey species were calculated using formula i/j*100, where ‘i’ represents the frequency of number of samples in which a specific prey occurs and ‘j’ represents the total frequency count of all prey species (Kruuk, 1989; Mukherjee *et al.,* 1994). Further, relative prey biomass consumed was calculated using formula D = (A*Y)/∑(A*Y)*100 where ‘A’ represents the RFO of each prey species and ‘Y’ represents weight of consumed prey in each faeces. ‘Y’ is calculated using Ackerman’s equation: Y = 1.980 + 0.035X, where X = mean body weight of a particular prey species (Ackerman *et al.,* 1984; Karanth and Sunquistt, 1995). The mean body weight of prey was taken from Harihar *et al.* (2011), Rathore (2015) and Upadhyaya *et al.* (2018) (Table 1). Two-way ANOVA was used to test any significant differences in prey RFO and biomass among sampled groups (spatial scale: ERTR_2015_ vs. WRTR_2015_, ERTR_2020_ vs. WRTR_2020_; temporal scale: ERTR_2015_ vs. ERTR_2020_, WRTR_2015_ vs. WRTR_2020_). Additionally, all the prey species data were categorized into three major classes: (a) large (≥60 kg), (b) medium (between 16 and 60 kg); and (c) small (≤15 kg) and absolute frequency of occurrence (AFO) was calculated for these classes using formula s_k_*100/*n*, where ‘s*_k_*’ is the number of samples containing class *k* and *n* is total number of faeces analysed (Harihar *et al.,* 2011). Any difference in AFO percentage in sampled groups were tested using chi-square analyses, followed by *f* test for pair wise comparison at spatial and temporal scales (mentioned above). For overall assessment of the habitat productivity, we compared mean NDVI values of three zones (WRTR, ERTR-core and ERTR-buffer) spatially using one-way ANOVA (with subsequent post hoc Tukey's HSD test) and temporally (between 2015 and 2020) using paired *t* test. All analyses were conducted using SPSS version 20 (IBM, 2011).

**Table 1:** Details of the various leopard diet parameters for all nice prey species identified in this study. Results are presented for percentage of relative frequency of occurrence (% RFO), and relative biomass of the consumed prey species in ERTR and WRTR for both 2015 and 2020, respectively

Prey species	Mean body weight of prey (in kg)	ERTR_2015_ (*n* = 86)		WRTR_2015_ (*n* = 76)		ERTR_2020_ (*n* = 53)		WRTR_2020_ (*n* = 89)	
		RFO (%)	Relative biomass (%)	RFO (%)	Relative biomass (%)	RFO (%)	Relative biomass (%)	RFO (%)	Relative biomass (%)
Livestock	250	5.81	12.50	0.00	0	1.89	4.32	0.00	0
Sambar	185	19.19	32.51	20.39	37.54	19.81	35.75	31.46	48.39
Nilgai	169	6.98	11.04	4.61	7.91	5.66	9.54	10.67	15.33
Chital	50	38.72	28.95	48.68	39.53	47.17	37.55	43.26	29.35
Wild pig	35	4.65	2.99	5.26	3.67	5.66	3.87	3.93	2.29
Hog deer	33	7.56	4.75	7.24	4.94	0.00	0	2.81	1.60
Langur	10	0.58	0.27	0.66	0.33	0.00	0	0.00	0
Hare	4	13.72	5.83	13.16	6.07	19.81	8.96	7.30	2.82
Indian peafowl	5	2.67	1.16	0.00	0	0.00	0	0.56	0.22

During hormone data analyses, the leopard fGCM and fT3M data (raw as well as log transformed) were assessed for normality using diagnostic plots (density plots) and Shapiro–Wilk test. Generalized linear models (GLMs) with log link and gamma distribution errors were used to explain the variation in fGCM and fT3M data. To assess any possible changes in fGCM and fT3M levels across spatial (ERTR vs WRTR) and temporal (2015 vs 2020) scales, an interaction term ‘Area*Year’ (as the tiger density in ERTR was lower in 2015 than in 2020, that may impact fGCM and fT3M levels) and the prey size class (large, medium and small, as prey size may impact fGCM and fT3M levels) were used as explanatory variables. Likelihood ratio test (LRT) was used to determine if the explanatory variables explain the data independently or in combination. Finally, post-hoc Tukey's HSD test was employed to assess any pair-wise differences in fGCM and fT3M levels for all sampled groups (mentioned above) and prey size classes. To evaluate the relationship of fGCM and fT3M with habitat productivity (NDVI values derived from sample buffers), we used two separate linear models (function ‘lm’). Additionally, we also performed a multivariate GLM including all three predictor variables (Area*Year, Prey size and NDVI change) and compared the resulting models with null model (Supplementary Material, Table S6) to discern the effect of each of the variable on fGCM and FT3M data. All analyses were conducted in R v4.1.1 (R Core Team, 2021) with the following packages: ‘ggpubr’ (Kassambara, 2020) and ‘multcomp’ (Hothorn *et al.,* 2008).

## Results

During the study period, a total of 564 large carnivore faeces was collected (*n* = 276: ERTR-172 and WRTR-104 samples in 2015 and *n* = 288: ERTR-178 and WRTR-110 samples in 2020) from the entire study area. After species confirmation, 324 leopard faecal samples were further processed for dietary and hormone analyses. The distribution of these samples was as followed: ERTR_2015_–92, WRTR_2015_–81, ERTR_2020_–60 and WRTR_2020_–91. However, prey species could be identified from 304 samples (93.82% success rate, ERTR_2015_–86, WRTR_2015_–76, ERTR_2020_–53 and WRTR_2020_–89, respectively; Table 1). The remaining samples (*n* = 20, 6.18%) contained damaged hairs for accurate species identification and were excluded from further dietary analyses. For hormone analyses, samples with > 80% IOM were discarded (*n* = 121) and finally 203 faecal hormone extracts (ERTR_2015_–56, WRTR_2015_–49, ERTR_2020_–42 and WRTR_2020_–56) were used in physiology analyses.

### Food habits of leopard

Overall, a total of nine prey species (large-bodied—Sambar, Nilgai and Livestock; medium-bodied—Chital, Wild pig and Hog deer and small-bodied—Langur, Hare and Peafowl) were detected. The large and medium-bodied prey species contributed 85.38% (RFO) of leopard diet whereas small prey species comprised only 14.62% (RFO). RFO of Chital (44.49%) and Sambar (22.71%) were highest followed by others (Table 1). However, biomass of Sambar was highest (38.55%), closely followed by chital (33.85%) (Table 1). Majority of the samples (*n* = 273, 89.8%) contained single prey species. All prey species except livestock (identified only in the ERTR) was found across all sampled groups. The rarefaction curve saturated beyond 40 samples within each group, and no new prey species was identified further (Supplementary Material, Fig. S2).

The two-way ANOVA analyses with all leopard prey species among the sampled groups (spatial: ERTR_2015_ vs. WRTR_2015_, ERTR_2020_ vs. WRTR_2020_; temporal: ERTR_2015_ vs. ERTR_2020_, WRTR_2015_ vs. WRTR_2020_) showed no significant differences in both RFO and biomass. However, overall comparisons within habitats across all compared groups indicated significant differences in both RFO and biomass (Supplementary Material, Table S5). Comparative analyses (Chi-square test with prey body-size groups) revealed large-sized prey frequencies differed significantly among sampled groups (χ^2^ = 8.62, *P* = 0.035). *F* test showed that at spatial scale, there were no significant differences in frequencies of different prey classes between ERTR_2015_ and WRTR_2015_ (*P* = 0.445, *P* = 0.476 and *P* = 1.00 for large, medium and small prey classes, respectively) (Table 2). However, compared to ERTR_2020,_ WRTR_2020_ showed significantly higher large-bodied (*P* = 0.018) and lower small-bodied prey species (*P* = 0.019). Temporally, the ERTR_2015_ and ERTR_2020_ showed no difference in prey class occurrences (*P* = 0.445 (large), *P* = 1.00 (medium) and *P* = 0.335 (small), whereas WRTR_2020_ showed a significant increase in large prey occurrences (*P* = 0.018) with no differences in medium and smaller-bodied prey classes (*P* = 0.156 and *P* = 0.238, respectively) compared to WRTR_2015_.

**Table 2:** Spatio-temporal comparison of differences in absolute frequency of prey occurrence in leopard diet at Rajaji Tiger Reserve, India. The comparisons were calculated using Fisher’s Exact test (2x2)

Prey category	Detection	Spatial comparison						Temporal comparison					
		ERTR_2015_	WRTR_2015_	*P*	ERTR_2020_	WRTR_2020_	*P*	ERTR_2015_	ERTR_2020_	*P*	WRTR_2015_	WRTR_2020_	*P*
Large	Yes	33.72	27.63	0.445	28.30	44.94	0.018^*^	33.72	28.30	0.445	27.63	44.94	0.018^*^
	No	66.28	72.37		71.70	55.06		66.28	71.70		72.37	55.06	
Medium	Yes	53.49	59.21	0.476	52.83	48.31	0.572	53.49	52.83	1.000	59.21	48.31	0.156
	No	46.51	40.79		47.17	51.69		46.51	47.17		40.79	51.69	
Small	Yes	12.79	13.16	1.000	18.87	6.74	0.019^*^	12.79	18.87	0.335	13.16	6.74	0.238
	No	87.21	86.84		81.13	93.26		87.21	81.13		86.84	93.26	

### NDVI values at spatio-temporal scales

**Figure 2:**
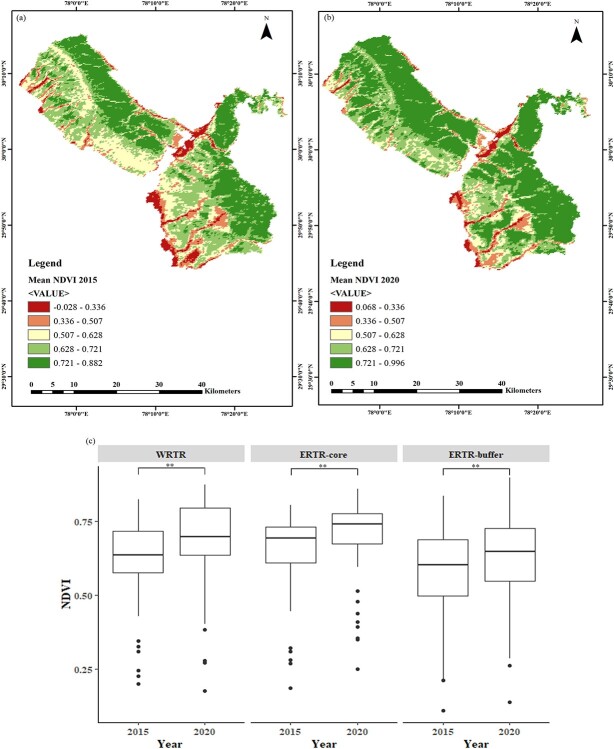
Assessment and comparison of habitat quality (through mean NDVI values) across core and buffer zones in two time points (winter 2015 and winter 2020). Panel (a) and (b) represents the mean NDVI gradients in 2105 and 2020, respectively. Panel (c) shows the temporal differences in mean NDVI values for WRTR, ERTR-core and ERTR-buffer areas. ^**^ indicate significance value at P < 0.005.

NDVI comparison using one-way ANOVA showed significant differences between zones (WRTR, ERTR-core and ERTR-buffer) in both 2015 (*F*(2,279) = 5.81, *P* = 0.003), as well as 2020 (*F*(2,279) = 7.74, *P* = 0.001), at spatial scale. Subsequent post hoc test showed that WRTR and ERTR-core zones did not differ significantly in their mean NDVI values in year 2015 (*P* = 0.47), as well as in 2020 (*P* = 0.93). However, ERTR-buffer zone continuously showed significantly lower NDVI values in both the years compared to WRTR (2015: *P* = 0.03, 2020: *P* = 0.001) and ERTR-core (2015: *P* = 0.004, 2020: *P* = 0.003) zones (Figure 2c). At temporal scale, paired *t* test showed that mean NDVI values improved significantly in year 2020 for WRTR (*t*(134) = 25.06, *P* < 0.0001), ERTR-core (*t*(66) = 9.39, *P* < 0.0001), as well as ERTR-buffer (*t*(79) = 12.45, *P* < 0.0001) zones compared to year 2015 (Figure 2c).

### Physiological responses of the sampled groups at spatial and temporal scales

**Figure 3:**
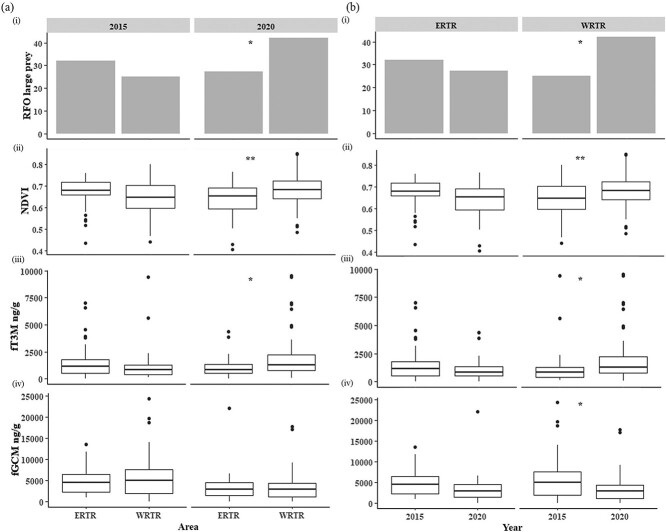
Spatio-temporal comparisons of the major variables (habitat variable—(i) large prey RFO, (ii) NDVI and physiological variable—(iii) fT3M and (iv) fGCM) used in this study. Panel (a) shows the temporal-scale differences and panel (b) indicate spatial differences. Significant differences are depicted by ^*^ (indicating *P* < 0.05) and ^**^ (indicating *P* < 0.005).

Likelihood ratio test selected the individual GLM explanatory variables (Area*Year and Prey size, respectively) over combined model as significant factors to explain the physiological response patterns (Supplementary Material, Table S3). GLM results with Area*Year model indicated year is a significant factor (*P* = 0.05) for fGCM data (Table 3). At spatial scale, both ERTR_2015_-WRTR_2015_ and ERTR_2020_-WRTR_2020_ comparisons showed no significant differences in mean fGCM levels (2015—*z* = 0.834, *P* = 0.838; and 2020—*z* = 0.253, *P* = 0.994) (post-hoc test, Supplementary Material, Table S2). However, temporal scale comparisons showed contrasting results where mean fGCM levels between ERTR_2015_ and ERTR_2020_ showed non-significant differences (*z* = −1.97, *P* = 0.2) but WRTR_2020_ had significantly low fGCM values than WRTR_2015_ (*z* = −2.73, *P* = 0.032) (Figure 3b(iv)). For fT3M data, GLM results showed that the area and year interaction factor (Area*Year) is a significant factor (Table 3). At spatial scale, the fT3M levels between ERTR_2015_-WRTR_2015_ showed no difference (*z* = −1.163, *P* = 0.65), but WRTR_2020_ fT3M levels were significantly higher than the ERTR_2020_ (*z* = 2.644, *P* = 0.041) (Figure 3a(iii)). At temporal scale, ERTR_2015_-ERTR_2020_ comparisons revealed no significant differences in fT3M levels (*z* = −1.303, *P* = 0.56) but WRTR_2020_ showed significantly higher values of fT3M than WRTR_2015_ (*z* = 2.602, *P* = 0.046) (Figure 3b(iii)).

**Table 3:** Results showing the association between the predictor (prey size categories and interaction of area and year) and response variables (fGCM and fT3M) based on GLM analyses. Models were fitted using log link and gamma error distributions

Response variables	Predictor variable	Level	Estimate	± SE	t value	Pr(>|*t*|)
fGCM	Area^*^Year	(Intercept)	162.256	78.035	2.079	0.0389^*^
		AreaWest	41.441	105.873	0.391	0.696
		Year	−0.076	0.039	−1.972	0.0500^*^
		AreaWest:Year	−0.020	0.052	−0.390	0.697
fGCM	Prey size (L, M and S)	(Intercept)	8.377	0.109	76.946	<2e-16^***^
		Prey size (Medium)	−0.042	0.142	−0.294	0.769
		Prey size (small)	0.341	0.214	1.592	0.113
fT3M	Area^*^Year	(Intercept)	133.090	96.622	1.377	0.170
		AreaWest	−356.293	131.091	−2.718	0.00718^**^
		Year	−0.062	0.048	−1.303	0.194
		AreaWest:Year	0.177	0.065	2.719	0.00716^**^
fT3M	Prey size (L, M and S)	(Intercept)	7.538	0.135	55.876	< 2e-16^***^
		Prey size (Medium)	−0.458	0.175	−2.612	0.00972^**^
		Prey size (small)	−0.306	0.266	−1.154	0.250

**Figure 4:**
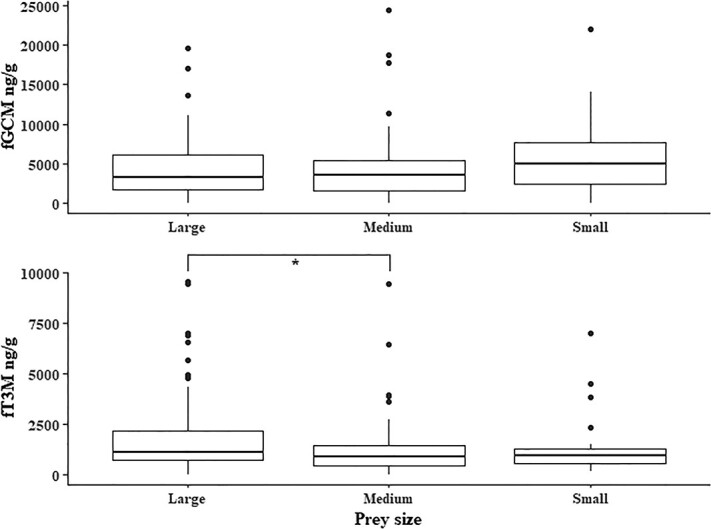
Comparison of (a) fGCM and (b) fT3M measures from faecal samples consisting large, medium and small body-sized prey. Significant differences are depicted by ^*^ (indicating *P* < 0.05).

GLM outputs with Preysize model indicated no significant differences between prey size class (large, medium and small body-sized prey) for fGCM levels (Table 3, Figure 4a). However, the fT3M levels showed strong relationship with prey size classes, where the fT3M levels from the leopard samples with large prey remains were higher than small prey class (*z* = 1.15, *P* = 0.5) and significantly higher than medium prey class (*z* = 2.61, *P* = 0.02) (Table 3, Figure 4b). Linear models showed a marginally significant positive association between leopard fGCM and habitat NDVI values (*t* value = 2.003, *F*(1, 201) = 4.01, *P* = 0.05) (Supplementary Material, Fig. S4a), and no significant association between leopard FT3M and habitat NDVI values (*t* value = −1.11, *F*(1, 201) = 1.23, *P* = 0.27) (Supplementary Material, Fig. S4b). GLM with all three variables (Area*Year, Prey size and NDVI change) shows that the interaction factor of Area and Year (indicating towards unique physiological responses of leopards in space and time) is included in the best models. NDVI and Prey size best explains the variation in the data only when included with Area*Year, but not alone, for both fGCM as well as fT3M (Supplementary Material, Table S6).

## Discussion

As expected, our results provide strong support for certain known patterns of tiger-leopard dominance dynamics. For example, we observed one such dominance hierarchy in the form of dietary niche separation within and between ERTR and WRTR at spatio-temporal scale. In 2015, our results show relatively higher chital and similar Sambar frequencies (two major prey species in the study area) in WRTR compared to ERTR (Table 1) but overall distribution of different prey size class (large, medium and small) remained similar. However, in 2020, the WRTR area showed significantly higher large-bodied and lower small-bodied prey species compared to ERTR (as hypothesized in this study). For ERTR (in 2020, spatially as well as temporally), relatively higher frequency of small-bodied prey in leopard diet can be explained through possible competitive spatial exclusion of leopards. During 2020 surveys majority of the leopard faecal samples were obtained from the buffer regions of the park, which are not prime habitats in terms of prey abundances (Harihar *et al.,* 2020). The southern buffer and Gohri range of ERTR (see Figure 1) supports lower prey densities (25.24 individuals/km^2^) than the core areas (39.23 individuals/km^2^) (Harihar *et al.,* 2020) and still hosts human settlements and livestock that exert heavy pressure on forest resources (Johnsingh and Joshua, 1994; Harihar *et al.,* 2014; Harihar *et al.,* 2020) and affects the ungulates population density by hindering the forest resource availability to wild prey species (Pozo *et al.,* 2021; Rasal *et al.,* 2022; Salvatori *et al.,* 2022). An alternate explanation behind such pattern could be leopard prey preferences for the medium to small bodied prey size class (Karanth and Sunquistt, 1995; Pokheral and Wegge, 2019), but the natural experimental setup (presence of tiger in ERTR and not in WRTR) allowed us to confirm the effects of competition. The results indicate that leopard consumption of medium to small sized prey in ERTR is not driven by their preference towards these particular classes but rather is a consequence of competition from tiger. Significantly high consumption of large body-sized prey in WRTR (in absence of tiger) supports that such pattern is driven by competition pressure. Earlier Vanak *et al.* (2013) has also suggested similar pattern of competition (instead of preference) driven dietary niche separation.

These patterns can also be explained through the results of the NDVI analyses presented here. Since the relocation of majority of the human settlements from the eastern and western RTR (from 2003 onwards) and creation of inviolate space the habitat productivity of the park has improved significantly (Harihar *et al.,* 2008). Our NDVI analyses supported this by showing significant increase in vegetation cover across the park (WRTR, ERTR-core and ERTR-buffer) (Figure 2a and b). We feel that the habitat improvement (and subsequent increase in prey density, Jhala *et al.,* 2015, 2020) along with no-competition from tigers has resulted in availability of large-bodied prey species in the WRTR. In the ERTR, despite the habitat improvement, the population faced competition pressure from tigers over years (tiger density doubled in 2020 in ERTR, Jhala *et al.,* 2020), resulting in their potential displacement to the buffer regions (sub-optimal habitats with medium-sized prey availability). This can be substantiated by observing the faecal location patterns in this study and earlier reports of tiger and leopard occupancy in the park (Harihar *et al.,* 2011; Rathore, 2015). Spatial projection of the confirmed ERTR leopard samples showed that most of the 2015 samples were distributed in the core areas whereas majority of the 2020 samples were from the buffer areas (southern boundaries of the core) (Figure 1). Given that our sampling was conducted in the same trails in the core region during both years it can be inferred that the leopard presence in ERTR core decreased in 2020, possibly due to competition from socially-dominant tigers (as seen in other Terai habitats, McDougal, 1988; Wegge *et al.,* 2009; Odden *et al.,* 2010; Kafley *et al.,* 2019; Thapa *et al.,* 2021). Further, the tiger and leopard population estimation data (Jhala *et al.,* 2020) also suggest more photographic captures of leopards at northern and southern boundaries of the ERTR-core during 2018–19. Earlier, Harihar *et al.* (2011) and Rathore (2015) also reported a decline in leopard density in ERTR-core and their activity hotspots were concentrated towards the peripheral areas of the park as compared with the tiger activity hotspots towards the core areas. On the contrary, leopard faeces were found across more uniformly in WRTR in both sampling periods in the absence of any competition from tigers in this side of the park.

The results of the physiological impacts of dietary differences and habitat productivity mirrored the earlier mentioned patterns. Our measurements of fGCM and fT3M largely followed the NDVI (proxy for habitat productivity) and prey size class patterns across ERTR and WRTR, respectively (Figure 3 a and b). At spatial scale, we did not observe any significant differences in both fGCM and fT3M between ERTR and WRTR in 2015. This pattern did not support our hypothesis where we expected higher fGCM measures (from tiger competition) and lower fT3M (from possibly reduced prey accessibility) in ERTR. However, in 2020, our data show slightly different pattern in physiological measures where the fT3M levels in WRTR was significantly higher than ERTR, but no difference was found in fGCM titers. When looked at temporal scale, these patterns provide contrasting patterns for the physiological measures. Within ERTR no significant difference in fGCM and fT3M values were found between 2015 and 2020 (similar to the spatial patterns) but we observed significant differences in both measures in WRTR. The temporal-scale data provide strong support to explain the patterns in the light of ecological variables (habitat productivity and resource availability). From the observed data, the influences of prey availability (associated with habitat productivity) on fT3M (nutritional hormone) can be inferred as these measures showed very similar patterns. If we consider WRTR (both 2015 and 2020) as an example, this habitat showed low NDVI values in 2015 (indicating low habitat productivity at that time due to still recovering phase after human relocation events) and corresponding lower frequency of large-body sized prey in the leopard faeces. However, during 2020 we observed drastic changes in both ecological factors where both NDVI and large prey frequencies have significantly increased. The fT3M measures exactly follow the same patterns (low in 2015 and significantly high in 2020), indicating a clear correlation between ecological variables and associated physiological responses (fT3M in this case). It is important to point out that absence of tiger in WRTR and stark differences in ecological variables in temporal scale made this inference easy compared to ERTR where more complex ecological interactions are seen. Apart from the other two ecological factors mentioned above, competition from tigers plays a major role in the patterns of physiological responses in ERTR resulting in different outcomes. Here, the data suggest decreasing habitat productivity and large prey frequency in leopard diet from 2015 to 2020, corroborating with a decreasing (but non-significant) trend in fT3M values between these two time periods. During 2015 the tiger density in ERTR was 2.90 (±0.87)/100 km^2^ (Jhala *et al.,* 2015) which increased to 8 (± =1.4)/100 km^2^ (Jhala *et al.,* 2020) during 2020, and the resulting increase in competition would explain the nutritional stress (low fT3M value) during 2020. Given the complex interactions among various ecological variables (habitat productivity, prey availability (in terms of size class) and various levels of competition pressures (from tigers)), we feel that it might take more time to observe significant differences in physiological parameters in ERTR. A similar study after 3 to 5 years may provide further clarification on any possible differences in fT3M values in ERTR.

Another possible factor that could play important role in leopard physiology in this landscape is intra-species competition. It is well-documented that the WRTR has experienced an increase in the leopard numbers during the study duration (Jhala *et al.,* 2021), possibly due to absence of wild tigers whereas increasing tiger population was associated with decreasing leopard density in ERTR-core (Harihar *et al.,* 2011). Such increase in population density in WRTR thus can cause some impact in leopard physiology. However, our data do not support this as any such intra-species competition should have resulted in higher fGCM (stress from competition) and lower fT3M (decrease nutritional status in a growing population) in WRTR in 2020 compared to 2015. Combined together, we can interpret that intra-species competition probably has no/less effects in the physiological pattern found during this study duration. However, it is important to point out that presenting the data as a whole (average value of fGCM from all samples collected) might also mask any possible local effects of such competitions. Future studies with fine-scale, grid-based sampling effort with leopard density information within each grid can help to address any effects of such competition.

One of the critical considerations in this study is the significant association of prey body size classes (large, medium and small) with fT3M measures. This was considered based on our available knowledge that large terrestrial predator distribution and abundance is significantly driven by higher biomass availability (Carbone *et al.,* 2011), and they are known to prefer large-bodied prey due to higher energy gains (Carbone *et al.,* 1999, 2007, 2011; Carbone and Gittleman, 2002; Radloff and Du Toit, 2004). Work on leopard energetics has also reported increased energy expenditure between meals when meal size from previous kill is large (Wilmers *et al.,* 2017). We used our data to test the effect of prey body size on fT3M measures, where leopard faeces with evidences of only specific body-size classes were identified and the fT3M measures were correlated (Figure 4b). In absence of any physiological validation of fT3M measures in leopard (see Mondol *et al.,* 2020 for tiger), this result can also be considered as biological validation of fT3M under field conditions.

The fGCM analyses showed slightly different patterns than the resource-driven fT3M data. Spatially, the ERTR and WRTR did not show any significant differences in fGCM levels in both 2015 and 2020 (Figure 3a(iv)), although 2020 fGCM values were relatively lower than 2015 (probably due to increased habitat productivity in 2020, at least for WRTR). However, at temporal scale, the ERTR and WRTR showed contrasting patterns (in terms of our hypotheses). In ERTR, we observed a lower value of fGCM in 2020 (non-significant) when compared to 2015. This is surprising when considered that in 2020 the NDVI as well as large prey proportion was lower in ERTR (with corresponding low fT3M indicating higher nutritional stress) (Figure 3b). Physiologically, this should result in high fGCM (Wasser *et al.,* 2011, 2017; Ayres *et al.,* 2012; Vynne *et al.,* 2014; Joly *et al.,* 2015; Dias *et al.,* 2017; McCormley *et al.,* 2018) values but the data show an opposite pattern. While it is difficult to point out the exact mechanism behind such pattern, one possible reason could be much less competition from tigers in the buffer areas (in 2020). This could be a preferred bargain for the spatially displaced leopards from the core areas of ERTR in 2020 (tiger density 8 (±1.4)/100 km^2^ in 2020) at the cost of better nutritional status. Such behavior has been earlier documented in many anti-predatory response studies (Ylönen *et al.,* 2006; Creel *et al.,* 2009; Wasser *et al.,* 2011). WRTR leopards, in absence of any competition exhibited expected physiological responses at temporal scale where we observed higher fGCM (and corresponding high nutritional stress/low fT3M) in 2015 and low nutritional stress (high fT3M) and low fGCM in 2020. Both these patterns are also corroborating with the habitat quality and prey size class information in respective time frames (Figure 3b). It is important to point out that recent studies have cautioned regarding careful interpretations of GC/fGCM data due to contrasting patterns of directionality in GC response to chronic stress (Dickens and Romero, 2013), where both increase and decrease in GC titer has been observed as a consequence of chronic stress. Therefore, this study provides strong evidence of combining additional hormones (such as T3 used here for nutritional stress) along with GC as biomarkers to reveal different physiological regulatory responses to the environment under different contexts. Multidisciplinary approaches, as used in this study, would also bring out more comprehensive and ecologically meaningful outcomes that can be used in making appropriate conservation interventions.

Finally, the unique natural experimental habitat scenario, spatio-temporal sampling strategy and the patterns of fGCM and fT3M levels bring out some important conservation perspectives for leopards. Our results suggest that from a physiological perspective prey body size (large, medium and small) and availability (driven by habitat productivity) directly affect the dominant–subordinate dynamics, which is further compounded by the competition between both species (resulting in competitive exclusion). This has critical conservation implications for areas surrounding majority of the tiger landscapes across India. India has recently declared doubling its tiger numbers (population estimate of 1411 (1165–1675) in 2006 to 2967 (2603–3346) in 2018) across the country (Jhala *et al.,* 2020). Such increase in tiger population is expected to increase pressure on sympatric leopard populations pushing them towards buffers or more sub-optimal habitats, further exacerbating chances of human–leopard conflict. Therefore, expansion of leopard monitoring and population estimation efforts to buffers, their management in the context of conflicts and understanding of local factors driving the changes in population pattern would be critical for their future conservation. Our results highlight the importance of good-quality habitats and prey base for this species and future conservation efforts should ensure availability of the same for their persistence. This study also emphasizes the importance of similar work on other carnivore guilds particularly in the context of the ongoing Anthropocene, which is affecting inter-species dynamics globally.

## Funding

This research was funded by Grant-in-Aid support from Wildlife Institute of India to Samrat Mondol. Samrat Mondol was supported by Department of Science and Technology INSPIRE Faculty Award (IFA12-LSBM-47).

## Conflict of Interest

The authors declare no conflict of interests.

## Data availability statement

The data will be available upon request.

## Authors’ contributions

Conceptualization, Data generation, Data curation, Formal analysis, Validation, Visualization, Writing-Original Draft, Writing-Review and Editing: [**Shiv Kumari Patel];** Data generation, Data curation, Writing-Review and Editing: [**Sourabh Ruhela, Suvankar Biswas and Supriya Bhatt];** Conceptualization, Writing-Review and Editing, Supervision: [**Bivash Pandav];** Conceptualization, Data Curation, Analysis, Resources, Writing-Original draft, Writing-Review and Editing, Supervision, Project administration, Funding acquisition: [**Samrat Mondol].**

## Supplementary Material

Web_Material_coad039

## References

[ref1] Ackerman BB , Lindzey FG, Hemker TP (1984) Cougar food habits in southern Utah. J Wildl Manage 48: 147. 10.2307/3808462.

[ref2] Ames EM , Gade MR, Nieman CL, Wright JR, Tonra CM, Marroquin CM, Tutterow AM, Gray SM (2020) Striving for population-level conservation: integrating physiology across the biological hierarchy. Conserv Physiol 8: coaa019. 10.1093/conphys/coaa019. 32274066 PMC7125044

[ref3] Andheria AP , Karanth KU, Kumar NS (2007) Diet and prey profiles of three sympatric large carnivores in Bandipur Tiger Reserve, India. J Zool 273: 169–175. 10.1111/j.1469-7998.2007.00310.x.

[ref4] Armitage KB (1991) Factors affecting corticosteroid concentrations in yellow-bellied marmots. Biochem Physiol 98: 47–54. 10.1016/0300-9629(91)90576-X. 1673377

[ref5] Ayres KL , Booth RK, Hempelmann JA, Koski KL, Emmons CK, Baird RW, Balcomb-Bartok K, Hanson MB, Ford MJ, Wasser SK (2012) Distinguishing the impacts of inadequate prey and vessel traffic on an endangered killer whale (*Orcinus orca*) population. PloS One 7: e36842. 10.1371/journal.pone.0036842. 22701560 PMC3368900

[ref6] Bahuguna A , Sahajpal V, Goyal SP, Mukherjee SK, Thakur V (2010) Species identification from guard hair of selected Indian mammals: a reference guide. Wildlife Institute of India, coaa019

[ref7] Behringer V , Deimel C, Hohmann G, Negrey J, Schaebs FS, Deschner T (2018) Applications for non-invasive thyroid hormone measurements in mammalian ecology, growth, and maintenance. Horm Behav. 105: 66–85. 10.1016/j.yhbeh.2018.07.011 30063897

[ref8] Berkmuller K , Bhatnagar S, Das B (1987) Pressure and dependency by local people on the resources of Rajaji National Park. Wildlife Institute of India, Dehradun

[ref9] Biswas S , Bhatt S, Paul S, Modi S, Ghosh T, Nigam P, Talukdar G, Pandav B, Mondol S (2019) A practive faeces collection protocol for multidisciplinary research in wildlife science. Curr Sci 116: 1878–1885. 10.18520/cs/v116/i11/1878-1885. https://www.currentscience.ac.in/Volumes/116/11/1878.pdf.

[ref10] Biswas S , Bhatt S, Sarkar D, Talukdar G, Pandav B, Mondol S (2022a) Assessing tiger corridor functionality with landscape genetics and modelling across Terai-Arc landscape, India. Conserv Genet 23: 949–966. 10.1007/s10592-022-01460-8.

[ref11] Biswas S , Kumar S, Bandhopadhyay M, Patel SK, Lyngdoh S, Pandav B, Mondol S (2022b) What drives prey selection? Assessment of tiger food habits across the Terai-Arc landscape, India. bioRxiv. 2022: 2022-07 10.1101/2022.07.20.500750.

[ref12] Broekhuis F , Cozzi G, Valeix M, Mcnutt JW, Macdonald DW (2013) Risk avoidance in sympatric large carnivores: reactive or predictive? J Anim Ecol 82: 1098–1105. 10.1111/1365-2656.12077. 23692142

[ref13] Carbone C , Gittleman JL (2002) A common rule for the scaling of carnivore density. Science 295: 2273–2276. 10.1126/science.1067994. 11910114

[ref14] Carbone C , Mace GM, Roberts SC, Macdonald DW (1999) Energetic constraints on the diet of terrestrial carnivores. Nature 402: 286–288. 10.1038/46266. 10580498

[ref15] Carbone C , Pettorelli N, Stephens PA (2011) The bigger they come, the harder they fall: body size and prey abundance influence predator–prey ratios. Biol Lett 7: 312–315. 10.1098/rsbl.2010.0996. 21106569 PMC3061189

[ref16] Carbone C , Teacher A, Rowcliffe JM (2007) The costs of carnivory. PLoS Biol 5: e22–e0368. 10.1371/journal.pbio.0050022. 17227145 PMC1769424

[ref17] Carlsson NOL , Jeschke JM, Holmqvist N, Kindberg J (2010) Long-term data on invaders: when the fox is away, the mink will play. Biol Invasions 12: 633–641. 10.1007/s10530-009-9470-z.

[ref18] Carter N , Jasny M, Gurung B, Liu J (2015) Impacts of people and tigers on leopard spatiotemporal activity patterns in a global biodiversity hotspot. Glob Ecol Conserv 3: 149–162. 10.1016/j.gecco.2014.11.013.

[ref19] Champion HG , Seth SK (2005) A revised survey of forest types of India. Dehradun, Natraj

[ref20] Clinchy M , Sheriff MJ, Zanette LY (2013) Predator-induced stress and the ecology of fear. Funct Ecol 27: 56–65. 10.1111/1365-2435.12007.

[ref21] Colwell R (2006) EstimateS: statistical estimation of species richness and shared species from samples. Version 9.1. User's Guide and application published at: http://purl.oclc.org/estimates.

[ref22] Cornhill KL , Kerley GIH (2020) Cheetah communication at scent-marking sites can be inhibited or delayed by predators. Behav Ecol Sociobiol 74: 1–10. 10.1007/s00265-020-2802-9.

[ref23] IBM Corp (2011) IBM SPSS Statistics for Windows Version 20.0, Armonk, NY: IBM Corp

[ref24] Creel S (2001) Social dominance and stress hormones. Trends Ecol Evol 16: 491–497. 10.1016/S0169-5347(01)02227-3.

[ref25] Creel S , Christianson D (2008) Relationships between direct predation and risk effects. Trends Ecol Evol 23: 194–201. 10.1016/j.tree.2007.12.004. 18308423

[ref26] Creel S , Christianson D, Liley S, Winnie JA (2007) Predation risk affects reproductive physiology and demography of elk. Science 315: 960. 10.1126/science.1135918. 17303746

[ref27] Creel S , Creel NM, Monfort SL (1996) Social stress and dominance. Nature 379: 212–212. 10.1038/379212a0.

[ref28] Creel S , Dröge E, M’soka J, Smit D, Becker M, Christianson D, Schuette P (2017) The relationship between direct predation and antipredator responses: a test with multiple predators and multiple prey. Ecology 98: 2081–2092. 10.1002/ecy.1885. 28475209

[ref29] Creel S , Winnie JA Jr, Christianson D (2009) Glucocorticoid stress hormones and the effect of predation risk on elk reproduction. Proc Natl Acad Sci 106: 12388–12393. 10.1073/pnas.0902235106. 19617549 PMC2718336

[ref30] Dantzer B , Fletcher QE, Boonstra R, Sheriff MJ (2014) Measures of physiological stress: a transparent or opaque window into the status, management and conservation of species? Conserv Physiol 2. 10.1093/conphys/cou023. PMC4732472 27293644

[ref31] Dias PAD , Coyohua-Fuentes A, Canales-Espinosa D, Chavira-Ramírez R, Rangel-Negrín A (2017) Hormonal correlates of energetic condition in mantled howler monkeys. Horm Behav 94: 13–20. 10.1016/j.yhbeh.2017.06.003. 28602941

[ref32] Dickens MJ , Romero LM (2013) A consensus endocrine profile for chronically stressed wild animals does not exist. Gen Comp Endocrinol. 191: 177–189. 10.1016/j.ygcen.2013.06.014 23816765

[ref33] Donadio E , Buskirk SW (2006) Diet, morphology, and interspecific killing in carnivora. Am Nat 167: 524–536. 10.1086/501033. 16670995

[ref34] Eales JG (1988) The influence of nutritional state on thyroid function in various vertebrates. Am Zool 28: 351–362. 10.1093/icb/28.2.351.

[ref35] Feit B , Feit A, Letnic M (2019) Apex predators decouple population dynamics between mesopredators and their prey. Ecosystems 22: 1606–1617. 10.1007/s10021-019-00360-2.

[ref36] Flier JS , Harris M, Hollenberg AN (2000) Leptin, nutrition, and the thyroid: the why, the wherefore, and the wiring commentary. J Clin Invest 105: 859–861. 10.1172/JCI9725. 10749565 PMC377492

[ref37] Gaynor KM , Brown JS, Middleton AD, Power ME, Brashares JS (2019) Landscapes of Fear: Spatial Patterns of Risk Perception and Response. Trends Ecol Evol 34: 355–368 30745252 10.1016/j.tree.2019.01.004

[ref38] Goswami S , Patel SK, Kadivar R, Tyagi PC, Malik PK, Mondol S (2021) Effects of a combined enrichment intervention on the behavioural and physiological welfare of captive Asiatic lions (*Panthera leo persica*). Appl Anim Behav Sci 236: 105222. 10.1016/j.applanim.2021.105222.

[ref39] Harihar A , Ghosh-Harihar M, MacMillan DC (2014) Human resettlement and tiger conservation—socio-economic assessment of pastoralists reveals a rare conservation opportunity in a human-dominated landscape. Biol Conserv 169: 167–175. 10.1016/j.biocon.2013.11.012.

[ref40] Harihar A , Ghosh-Harihar M, Macmillan DC (2018) Losing time for the tiger *Panthera tigris*: delayed action puts a globally threatened species at risk of local extinction. Oryx 52: 78–88. 10.1017/S0030605317001156.

[ref41] Harihar A , Pandav B (2012) Influence of connectivity, wild prey and disturbance on occupancy of tigers in the human-dominated western Terai Arc landscape. PloS One 7: e40105. 10.1371/journal.pone.0040105. 22792220 PMC3390357

[ref42] Harihar A , Pandav B, Ghosh-Harihar M, Goodrich J (2020) Demographic and ecological correlates of a recovering tiger (*Panthera tigris*) population: lessons learnt from 13-years of monitoring. Biol Conserv 252: 108848. 10.1016/j.biocon.2020.108848.

[ref43] Harihar A , Pandav B, Goyal SP (2009a) Responses of tiger (*Panthera tigris*) and their prey to removal of anthropogenic influences in Rajaji National Park, India. Eur J Wildl Res 55: 97–105. 10.1007/s10344-008-0219-2.

[ref44] Harihar A , Pandav B, Goyal SP (2011) Responses of leopard *Panthera pardus* to the recovery of a tiger (*Panthera tigris*) population. J Appl Ecol 48: 806–814. 10.1111/j.1365-2664.2011.01981.x.

[ref45] Harihar A , Prasad DL, Ri C, Pandav B, Goyal SP (2009b) Losing ground: *Tigers panthera tigris* in the north-western Shivalik landscape of India. Oryx 43: 35–43. 10.1017/S0030605307072043.

[ref46] Hayward MW , Slotow R (2009) Temporal partitioning of activity in large African carnivores: tests of multiple hypotheses. African J Wildl Res 39: 109–125. 10.3957/056.039.0207.

[ref47] Hothorn T , Bretz F, Westfall P (2008) Simultaneous inference in general parametric models. Biom J 50: 346–363. 10.1002/bimj.200810425. 18481363

[ref48] Jhala YV , Qureshi Q, Gopal R (eds) (2015) The status of tigers copredators and prey in India, 2014. In National Tiger Conservation Authority. Government of India, New Delhi, and Wildlife Institute of India, Dehradun

[ref49] Jhala YV , Qureshi Q, Nayak AK (eds) (2020) Status of tigers, copredators and prey in India, 2018. In National Tiger Conservation Authority. Government of India, New Delhi, and Wildlife Institute of India, Dehradun

[ref50] Jhala YV , Qureshi Q, Yadav SP (2021) Status of leopards, co-predators, and megaherbivores in India, 2018. In National Tiger Conservation Authority. Government of India, New Delhi, and Wildlife Institute of India, Dehradun, ISBN-81-85496-56-0

[ref51] Johnsingh AJT (2006) Status and conservation of the tiger in Uttaranchal, Northern India. Ambio 35: 135–137. 10.1579/0044-7447(2006)35[135:SACOTT]2.0.CO;2. 16846203

[ref52] Johnsingh AJT , Joshua J (1994) Conserving Rajaji and Corbett National Parks—the elephant as a flagship species. Oryx 28: 135–140. 10.1017/S0030605300028453. http://journals.cambridge.org.

[ref53] Johnsingh AJT , Negi AS (2003) Status of tiger and leopard in Rajaji-Corbett Conservation Unit, northern India. Biol Conserv 111: 385–393. 10.1016/S0006-3207(02)00307-5.

[ref54] Johnsingh AJT , Prasad SN, Goyal SP (1990) Conservation status of the Chila-Motichur corridor for elephant movement in Rajaji-Corbett National Parks area, India. Biol Conserv 51: 125–138. 10.1016/0006-3207(90)90107-Z.

[ref55] Joly K , Wasser SK, Booth R (2015) Non-invasive assessment of the interrelationships of diet, pregnancy rate, group composition, and physiological and nutritional stress of barren-ground caribou in late winter. PloS One 10: e0127586. 10.1371/journal.pone.0127586. 26061003 PMC4464525

[ref56] Kafley H , Lamichhane BR, Maharjan R, Khadka M, Bhattarai N, Gompper ME (2019) Tiger and leopard co-occurrence: intraguild interactions in response to human and livestock disturbance. Basic Appl Ecol 40: 78–89. 10.1016/j.baae.2019.07.007.

[ref57] Karanth KU , Sunquist ME (2000) Behavioural correlates of predation by tiger (*Panthera tigris*), leopard (*Panthera pardus*) and dhole (*Cuon alpinus*) in Nagarahole, India. J Zool 250: 255–265. 10.1111/j.1469-7998.2000.tb01076.x.

[ref58] Karanth KU , Sunquistt ME (1995) Prey selection by tiger, leopard and dhole in tropical forests. J Anim Ecol 64: 439–450. 10.2307/5647.

[ref59] Kassambara A (2020) ggpubr: “ggplot2”. Based Publication Ready Plots. https://CRAN.R-project.org/package=ggpubr

[ref60] Kruuk H (1989) The social badger. Ecology and behaviour of a group-living carnivore (Meies meles). Oxford University Press, Oxford–New York–Tokyo

[ref61] Kumar U , Awasthi N, Qureshi Q, Jhala YV (2019) Do conservation strategies that increase tiger populations have consequences for other wild carnivores like leopards? Sci Rep 9: 14673. 10.1038/s41598-019-51213-w. 31604995 PMC6789119

[ref62] Lamanna JA , Martin TE (2016) Costs of fear: behavioural and life-history responses to risk and their demographic consequences vary across species. Ecol Lett 19: 403–413. 10.1111/ele.12573. 26900087

[ref63] Laurenson MK (1994) High juvenile mortality in cheetahs (*Acinonyx jubatus*) and its consequences for maternal care. J Zoo 234: 387–408. 10.1111/j.1469-7998.1994.tb04855.x.

[ref64] Linnell JDC , Strand O (2000) Interference interactions, co-existence and conservation of mammalian carnivores. Divers Distrib 6: 169–176. 10.1046/j.1472-4642.2000.00069.x. http://www.blackwell-science.com/ddi.

[ref65] López-Bao JV , Mattisson J, Persson J, Aronsson M, Andrén H (2016) Tracking neighbours promotes the coexistence of large carnivores. Sci Rep 6. 10.1038/srep23198. PMC4793264 26979573

[ref66] MacLeod KJ , Krebs CJ, Boonstra R, Sheriff MJ (2018) Fear and lethality in snowshoe hares: the deadly effects of non-consumptive predation risk. Oikos 127: 375–380. 10.1111/oik.04890.

[ref67] Magurran AE (2004) Measuring Biological Diversity. Blackwell Publishing, Oxford

[ref68] McCormley MC , Champagne CD, Deyarmin JS, Stephan AP, Crocker DE, Houser DS, Khudyakov JI (2018) Repeated adrenocorticotropic hormone administration alters adrenal and thyroid hormones in free-ranging elephant seals. Conserv Physiol 6. 10.1093/conphys/coy040. PMC6048993 30034809

[ref69] McDougal C (1988) Leopard and tiger interactions at Royal Chitwan National park. Nepal J Bombay Nat Hist Soc 85: 609–611. https://www.biodiversitylibrary.org/item/191948.

[ref70] Merkle JA , Stahler DR, Smith DW (2009) Interference competition between gray wolves and coyotes in Yellowstone National Park. Can J Zool 87: 56–63. 10.1139/Z08-136.

[ref71] Mitchell BD , Banks PB (2005) Do wild dogs exclude foxes? Evidence for competition from dietary and spatial overlaps. Austral Ecol 30: 581–591. 10.1111/j.1442-9993.2005.01473.x.

[ref72] Mondal K (2012) Response of leopards to re-introduced tigers in Sariska Tiger Reserve, Western India. Int J Biodivers Conserv 4. 10.5897/ijbc12.014.

[ref73] Mondol S , Booth RK, Wasser SK (2020) Fecal stress, nutrition and reproductive hormones for monitoring environmental impacts on tigers (*Panthera tigris*). Conserv Physiol 8: coz091. 10.1093/conphys/coz091. PMC6955020 31942242

[ref74] Mondol S , Kumar NS, Gopalaswamy A, Sunagar K, Karanth KU, Ramakrishnan U (2015) Identifying species, sex and individual tigers and leopards in the Malenad-Mysore Tiger landscape, Western Ghats, India. Conserv Genet Resour 7: 353–361. 10.1007/s12686-014-0371-9.

[ref75] Mukherjee S , Goyal SP, Chellam R (1994) Standardisation of scat analysis techniques for leopard (*Panthera pardus*) in Gir National Park, Western India. Mammalia 58: 139–143. 10.1515/mamm.1994.58.1.139.

[ref76] Newsome TM , Greenville AC, Ćirović D, Dickman CR, Johnson CN, Krofel M, Letnic M, Ripple WJ, Ritchie EG, Stoyanov S et al. (2017) Top predators constrain mesopredator distributions. Nat Commun 8. 10.1038/ncomms15469. PMC5457510 28534486

[ref77] Odden M , Wegge P, Fredriksen T (2010) Do tigers displace leopards? If so, why? Ecol Res 25: 875–881. 10.1007/s11284-010-0723-1.

[ref78] Palme R (2019). Non-invasive measurement of glucocorticoids: Advances and problems. Physio Behav 199: 229–243. Elsevier Inc. 10.1016/j.physbeh.2018.11.021, 229, 243 30468744

[ref79] Palomares F , Caro TM (1999) Interspecific killing among mammalian carnivores. Am Nat 153: 492–508. 10.1086/303189. 29578790

[ref80] Parker KL , Barboza PS, Gillingham MP (2009) Nutrition integrates environmental responses of ungulates. Func Ecol 23: 57–69. 10.1111/j.1365-2435.2009.01528.x.

[ref81] Patel SK , Biswas S, Goswami S, Bhatt S, Pandav B, Mondol S (2021) Effects of faecal inorganic content variability on quantifying glucocorticoid and thyroid hormone metabolites in large felines: implications for physiological assessments in free-ranging animals. Gen Comp Endocrinol 310: 113833. 10.1016/j.ygcen.2021.113833. 34089705

[ref82] Périquet S , Fritz H, Revilla E (2015) The lion king and the hyaena queen: large carnivore interactions and coexistence. Biol Rev 90: 1197–1214. 10.1111/brv.12152. 25530248

[ref83] Pettorelli N , Ryan S, Mueller T, Bunnefeld N, Jedrzejewska B, Lima M, Kausrud K (2011) The Normalized Difference Vegetation Index (NDVI): unforeseen successes in animal ecology. Climate Res 46: 15–27. 10.3354/cr00936.

[ref84] Pettorelli N , Vik JO, Mysterud A, Gaillard JM, Tucker CJ, Stenseth NC (2005a) Using the satellite-derived NDVI to assess ecological responses to environmental change. Trends Ecol Evol 20: 503–510. 10.1016/j.tree.2005.05.011. 16701427

[ref85] Pettorelli N , Weladji RB, Holand Ø, Mysterud A, Breie H, Stenseth NC (2005b) The relative role of winter and spring conditions: linking climate and landscape-scale plant phenology to alpine reindeer body mass. Biol Lett 1: 24–26. 10.1098/rsbl.2004.0262. 17148119 PMC1629060

[ref86] Pokheral CP , Wegge P (2019) Coexisting large carnivores: spatial relationships of tigers and leopards and their prey in a prey-rich area in lowland Nepal. Ecosci 26: 1–9. 10.1080/11956860.2018.1491512.

[ref87] Pozo RA , Cusack JJ, Acebes P, Malo JE, Traba J, Iranzo EC, Morris-Trainor Z, Minderman J, Bunnefeld N, Radic-Schilling S et al. (2021) Reconciling livestock production and wild herbivore conservation: challenges and opportunities. Trends Ecol Evol 36: 750–761. 10.1016/j.tree.2021.05.002. 34103191

[ref88] R Core Team (2021) R: A language and environment for statistical computing. R Foundation for Statistical Computing, Vienna, Austria. https://www.R-project.org/.

[ref89] Radloff FGT , Du Toit JT (2004) Large predators and their prey in a southern African savanna: a predator’s size determines its prey size range. J Anim Ecol 73: 410–423. 10.1111/j.0021-8790.2004.00817.x.

[ref90] Ramesh T , Kalle R, Downs CT (2017) Staying safe from top predators: patterns of co-occurrence and inter-predator interactions. Behav Ecol Sociobiol 71. 10.1007/s00265-017-2271-y.

[ref91] Rasal V , Dhakad M, Khandal D, Chandrawal K (2022) Assessment of livestock grazing pressure in key tiger habitat in a semi-arid landscape in Western India. Trop Ecol 63: 644–649. 10.1007/s42965-022-00241-1.

[ref92] Rathore SR (2015) Response of leopard (Panthera pardus fusca) in varying density of tiger (Panthera tigris tigris) in Rajaji National Park, Uttarakhand. Wildlife Institute of India, Dehradun

[ref93] Ripple WJ , Estes JA, Beschta RL, Wilmers CC, Ritchie EG, Hebblewhite M, Berger J, Elmhagen B, Letnic M, Nelson MP et al. (2014) Status and ecological effects of the world's largest carnivores. Science 343. 10.1126/science.1241484. 24408439

[ref94] Ritchie EG , Elmhagen B, Glen AS, Letnic M, Ludwig G, McDonald RA (2012) Ecosystem restoration with teeth: what role for predators? Trends Ecol Evol 27: 265–271. 10.1016/j.tree.2012.01.001. 22321653

[ref95] Ritchie EG , Johnson CN (2009) Predator interactions, mesopredator release and biodiversity conservation. Ecol Lett 12: 982–998. 10.1111/j.1461-0248.2009.01347.x. 19614756

[ref96] Roy A (2003) An insight into socio-economic aspects and psychology of rehabilitated gujjar population in Pathri and Gaindikhatta relocation sites. Wildlife Institute of India, Dehradun

[ref97] Salvatori M , Oberosler V, Augugliaro C, Krofel M, Rovero F (2022) Effects of free-ranging livestock on occurrence and inter-specific interactions of a mammalian community. Ecol Appl 32: e2644. 10.1002/eap.2644. 35471769 PMC9788037

[ref98] Sapolsky RM (1983) Endocrine aspects of social instability in the olive baboon (*Papio anubis*). Am J Primatol 5: 365–379. 10.1002/ajp.1350050406. 31986851

[ref99] Seidensticker J (1976) On the ecological separation between tigers and leopards. Biotropica 8: 225–234. 10.2307/2989714.

[ref100] Seidensticker J , Sunquist ME, McDougal C (1990) Leopards living at the edge of the Royal Chitwan National Park, Nepal. In JC Daniel, JS Serrao, eds, Conservation in developing countries: Problems and prospects. Proceedings of the Centenary Seminar of the Bombay Natural History Society and Oxford University Press, Bombay, pp. 415–423

[ref101] Sheriff MJ , Peacor SD, Hawlena D, Thaker M (2020) Non-consumptive predator effects on prey population size: a dearth of evidence. J Anim Ecol 89: 1302–1316. 10.1111/1365-2656.13213. 32215909

[ref102] Sopinka NM , Patterson LD, Redfern JC, Pleizier NK, Belanger CB, Midwood JD, Crossin GT, Cooke SJ (2015) Manipulating glucocorticoids in wild animals: basic and applied perspectives. Conserv Physiol 3: cov031. 10.1093/conphys/cov031. 27293716 PMC4778459

[ref103] Steinmetz R , Seuaturien N, Chutipong W (2013) Tigers, leopards, and dholes in a half-empty forest: assessing species interactions in a guild of threatened carnivores. Biol Conserv 163: 68–78. 10.1016/j.biocon.2012.12.016.

[ref104] Suraci JP , Clinchy M, Dill LM, Roberts D, Zanette LY (2016) Fear of large carnivores causes a trophic cascade. Nat Commun 7: 1–7. 10.1038/ncomms10698. PMC4766389 26906881

[ref105] Szott ID , Pretorius Y, Ganswindt A, Koyama NF (2020) Normalized difference vegetation index, temperature and age affect faecal thyroid hormone concentrations in free-ranging African elephants. Conserv Physiol 8. 10.1093/conphys/coaa010. PMC7297438 32577285

[ref106] Thapa K , Malla S, Subba SA, Thapa GJ, Lamichhane BR, Subedi N, Dhakal M, Acharya KP, Thapa MK, Neupane P et al. (2021) On the tiger trails: leopard occupancy decline and leopard interaction with tigers in the forested habitat across the Terai Arc landscape of Nepal. Glob Ecol Conserv 25: e01412. 10.1016/j.gecco.2020.e01412.

[ref107] Upadhyaya SK , Musters CJM, Lamichhane BR, de Snoo GR, Thapa P, Dhakal M, Karmacharya D, Shrestha PM, de Iongh HH (2018) An insight into the diet and prey preference of tigers in Bardia National Park, Nepal. Trop Conserv Sci 11: 1–9. 10.1177/1940082918799476.

[ref108] Van Meter PE , French JA, Dloniak SM, Watts HE, Kolowski JM, Holekamp KE (2009) Fecal glucocorticoids reflect socio-ecological and anthropogenic stressors in the lives of wild spotted hyenas. Horm Behav 55: 329–337. 10.1016/j.yhbeh.2008.11.001. 19056392 PMC2987620

[ref109] Vanak AT , Fortin D, Thaker M, Ogden M, Owen C, Greatwood S, Slotow R (2013) Moving to stay in place: behavioral mechanisms for coexistence of African large carnivores. Ecology 94: 2619–2631. 10.1890/13-0217.1. 24400513

[ref110] Vynne C , Booth RK, Wasser SK (2014) Physiological implications of landscape use by free-ranging maned wolves (*Chrysocyon brachyurus*) in Brazil. J Mammal 95: 696–706. 10.1644/12-MAMM-A-247.

[ref111] Wasser SK , Cristóbal-Azkarate J, Booth RK, Hayward L, Hunt K, Ayres K, Vynne C, Gobush K, Canales-Espinosa D, Rodríguez-Luna E (2010) Non-invasive measurement of thyroid hormone in feces of a diverse array of avian and mammalian species. Gen Comp Endocrinol 168: 1–7. 10.1016/j.ygcen.2010.04.004. 20412809

[ref112] Wasser SK , Keim JL, Taper ML, Lele SR (2011) The influences of wolf predation, habitat loss, and human activity on caribou and moose in the Alberta oil sands. Front Ecol Environ 9: 546–551. 10.1890/100071.

[ref113] Wasser SK , Lundin JI, Ayres K, Seely E, Giles D, Balcomb K, Hempelmann J, Parsons K, Booth R (2017) Population growth is limited by nutritional impacts on pregnancy success in endangered southern resident killer whales (Orcinus orca). PloS One 12: e0179824. 10.1371/journal.pone.0179824. 28662095 PMC5491047

[ref114] Wasser SK , Thomas R, Nair PP, Guidry C, Southers J, Lucas JWDE, Monfort SL (1993) Effects of dietary fibre on faecal steroid measurements in baboons (*Papio cynocephalus cynocephalus*). Reproduction 97: 569–574. 10.1530/jrf.0.0970569. 8388960

[ref115] Wegge P , Odden M, Pokharel CP, Storaas T (2009) Predator–prey relationships and responses of ungulates and their predators to the establishment of protected areas: a case study of tigers, leopards and their prey in Bardia National Park, Nepal. Biol Conserv 142: 189–202. 10.1016/j.biocon.2008.10.020.

[ref116] Wilmers CC , Isbell LA, Suraci JP, Williams TM (2017) Energetics-informed behavioral states reveal the drive to kill in African leopards. Ecosphere 8. 10.1002/ecs2.1850.

[ref117] Ylönen H , Eccard JA, Jokinen I, Sundell J (2006) Is the antipredatory response in behaviour reflected in stress measured in faecal corticosteroids in a small rodent? Behav Ecol Sociobiol 60: 350–358. 10.1007/s00265-006-0171-7.

