# Supplementary material for: The cost of sympatry: spatio-temporal patterns in leopard dietary and physiological responses to tiger competition gradient in Rajaji Tiger Reserve, Uttarakhand, India
Source: Conserv Physiol. 2023 May 29;11(1):coad039. doi: 10.1093/conphys/coad039 (PMC10660413; doi:10.1093/conphys/coad039)

Supplementary Material

Supplementary Table 1: Details of the faecal hormone assays conducted for leopards in this study.

Hormone	Assay method	Dilution	Slope (R^2)	Inter-assay CV	Intra-assay CV	Cross-reactivity
Corticosterone (Leopard pool)	EIA	1:120	1.09 (0.98)	10.35	7.15	100% with corticosterone, 12.30% with Desoxycorticosterone, 2.30% with Tetrahydrocorticosterone and <1% with Aldosterone, Cortisol, Progesterone, Dexamethasone, Corticosterone-21-Hemisuccinate, Cortisone and Estradiol
Triiodothyronine (Leopard Pool)	EIA	1:7.5	1.02 (0.99)	7.86	8.36	100% with T3, 0.88% with thyroxine and less than 0.1% with reverse T3 (3,3',5'-Triiodo-L-thyronine)

Supplementary Table 2: Results of Post hoc pair wise comparisons.

	**Post hoc (Tukey HSD)**	**Estimate**	**Std. Error**	**z value**	**Pr(>|z|)**
fGCM (Area*Year)	$ERTR_{2020}$ - $ERTR_{2015}$	-0.382	0.193	-1.972	0.198
	$WRTR_{2020}$ - $WRTR_{2015}$	-0.484	0.177	-2.729	0.0317 *
	$WRTR_{2015}$ - $ERTR_{2015}$	0.151	0.181	0.834	0.838
	$WRTR_{2020}$ - $ERTR_{2020}$	0.048	0.190	0.253	0.994
fGCM (Prey size)	Medium - Large	-0.042	0.142	-0.294	0.952
	Small - Large	0.341	0.214	1.592	0.243
	Small - Medium	0.383	0.206	1.863	0.146
fT3M (Area*Year)	$ERTR_{2020}$ - $ERTR_{2015}$	-0.312	0.240	-1.303	0.560
	$WRTR_{2020}$ - $WRTR_{2015}$	0.571	0.220	2.602	0.0456 *
	$WRTR_{2015}$ - $ERTR_{2015}$	-0.260	0.224	-1.163	0.649
	$WRTR_{2020}$ - $ERTR_{2020}$	0.623	0.236	2.644	0.0408 *
fT3M (Prey size)	Medium - Large	-0.458	0.175	-2.612	0.024 *
	Small - Large	-0.306	0.266	-1.154	0.475
	Small - Medium	0.152	0.255	0.596	0.819

Supplementary Table 3: Results of Likelihood ratio test (LRT).

**Likelihood ratio test**	
**Models**	**Pr(>Chi)**
fGCM ~ Prey size	0.006**
fGCM ~ Prey size + Area * Year	
fGCM ~ Area*Year	0.117
fGCM ~ Prey size + Area * Year	
fT3M ~ Prey size	0.092
fT3M ~ Prey size + Area * Year	
fT3M ~ Area*Year	0.164
fT3M ~ Prey size + Area * Year	

Supplementary Table 4: Influence of NDVI values on fGCM and fT3M levels in leopards based on linear models.

Response	Predictor	Estimate	±SE	t value	Pr (>|t|)
fGCM	(Intercept)	-1014	2764	-0.367	0.714
	NDVI	8309	4149	2.003	0.0465*
fT3M	(Intercept)	2630	1075	2.447	0.0153
	NDVI	-1786	1613	-1.107	0.2697

lm (formula = fGCM~ NDVI, data); lm (formula = fT3M~ NDVI, data).

Supplementary Table 5: Results showing the output of two-way ANOVA, showing prey RFO and biomass comparison within habitat across all compared groups.

S.No.	variable	Groups compared	F statistic	P-value
1	Prey RFO	$ERTR_{2015}$ vs. $WRTR_{2015}$	40.39	0.00001
2	Prey RFO	$ERTR_{2020}$ vs. $WRTR_{2020}$	21.49	0.0001
3	Prey RFO	$ERTR_{2015}$ vs. $ERTR_{2020}$	31.16	0.00003
4	Prey RFO	$WRTR_{2015}$ vs. $WRTR_{2020}$	30.74	0.00003
5	Prey biomass	$ERTR_{2015}$ vs. $WRTR_{2015}$	19.49	0.0001
6	Prey biomass	$ERTR_{2020}$ vs. $WRTR_{2020}$	24.61	0.00007
7	Prey biomass	$ERTR_{2015}$ vs. $ERTR_{2020}$	29.92	0.00003
8	Prey biomass	$WRTR_{2015}$ vs. $WRTR_{2020}$	27.92	0.00004

Supplementary Table 6: Results showing the output of multivariate GLM, showing the best selected models.

**Models tested for fGCM**	**(Intercept)**	**family**	**df**	**logLik**	**AICc**	**delta**	**weight**
**Area*Year + mean NDVI**	153.827	Gamma(log)	6.00	-1902.531	3817.491	**0.000**	**0.616**
**Area*Year**	192.982	Gamma(log)	5.00	-1904.893	3820.091	**2.600**	**0.168**
Area*Year + mean NDVI + Prey size	161.675	Gamma(log)	9.00	-1901.083	3821.098	3.607	0.101
mean NDVI	7.116	Gamma(log)	3.00	-1908.311	3822.742	5.251	0.045
Area*Year + Prey size	198.106	Gamma(log)	8.00	-1903.325	3823.392	5.900	0.032
Null	8.409	Gamma(log)	2.00	-1909.977	3824.014	6.522	0.024
mean NDVI + Prey size	7.160	Gamma(log)	6.00	-1906.692	3825.813	8.322	0.010
Prey size	8.573	Gamma(log)	5.00	-1908.458	3827.220	9.728	0.005
**Models tested for fT3M**	**(Intercept)**	**family**	**df**	**logLik**	**AICc**	**delta**	**weight**
**Area*Year + mean NDVI + Prey size**	167.658	Gamma(log)	9.00	-1663.959	3346.851	**0.000**	**0.431**
**Area*Year + mean NDVI**	173.442	Gamma(log)	6.00	-1667.307	3347.042	**0.191**	**0.391**
Area*Year + Prey size	136.794	Gamma(log)	8.00	-1666.385	3349.513	2.662	0.114
Area*Year	143.632	Gamma(log)	5.00	-1670.437	3351.178	4.327	0.049
Prey size	6.737	Gamma(log)	5.00	-1672.294	3354.893	8.042	0.008
mean NDVI + Prey size	7.662	Gamma(log)	6.00	-1671.347	3355.123	8.272	0.007
mean NDVI	8.296	Gamma(log)	3.00	-1678.505	3363.130	16.279	0.000
Null	7.278	Gamma(log)	2.00	-1679.688	3363.437	16.586	0.000

Area*Year	Interaction factor of Space and Time, categorizing overall hormone data in four group W2015, W2020, E2015 and E2020
mean NDVI	in 2km radius buffer around each faecal sample
Prey size	Large, Medium and Small size prey consumed

Supplementary Figure 1: Parallelism and accuracy plots for faecal corticosterone and T3 EIA assays.

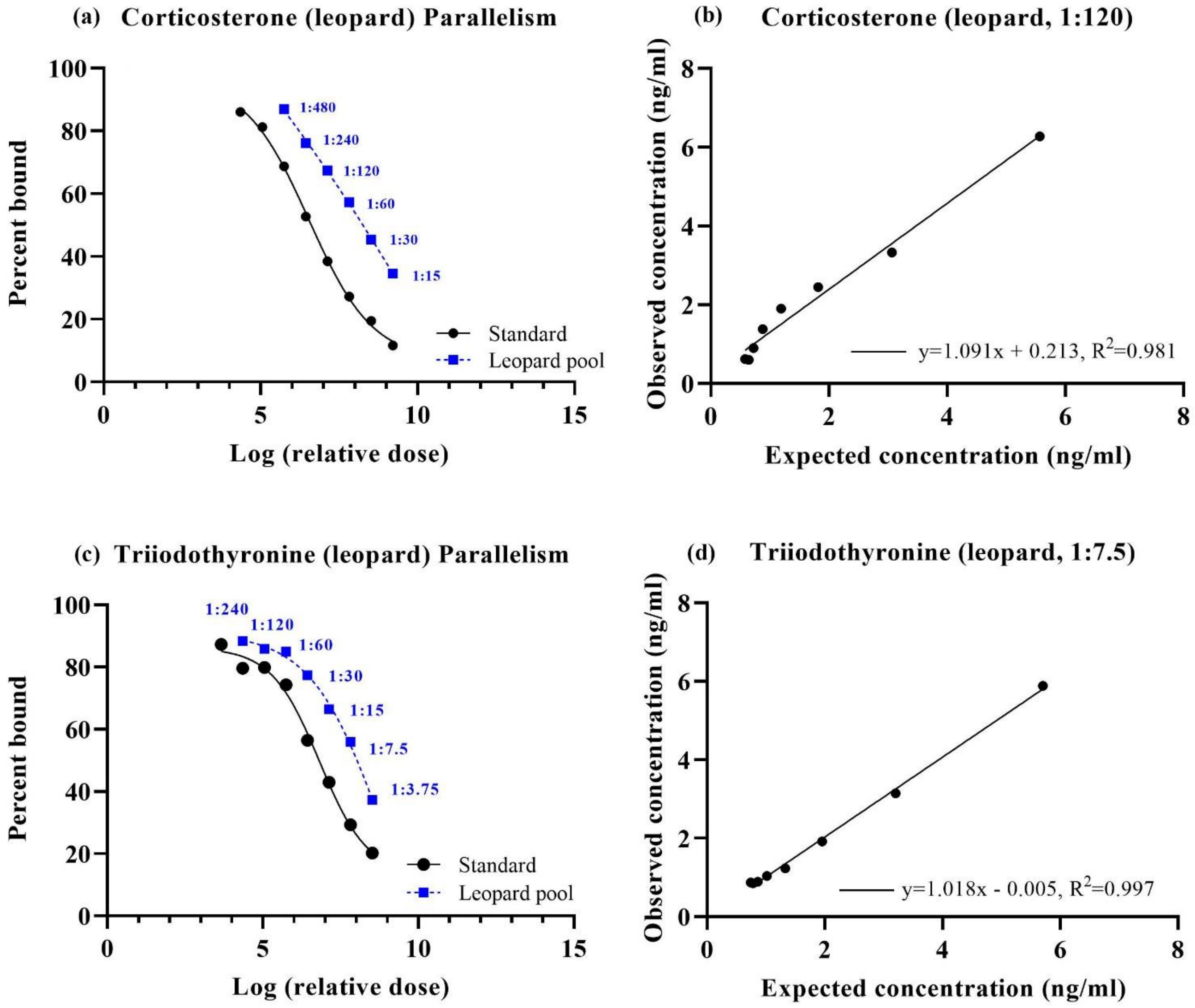

Supplementary Figure 2: The rarefaction curves indicating sample sufficiency for leopard food habit assessments. Different line types represent separate spatial (ERTR and WRTR) and temporal (2015 and 2020) sample combinations. The curves stabilize at a sample size of ~40, represented by dashed blue line.

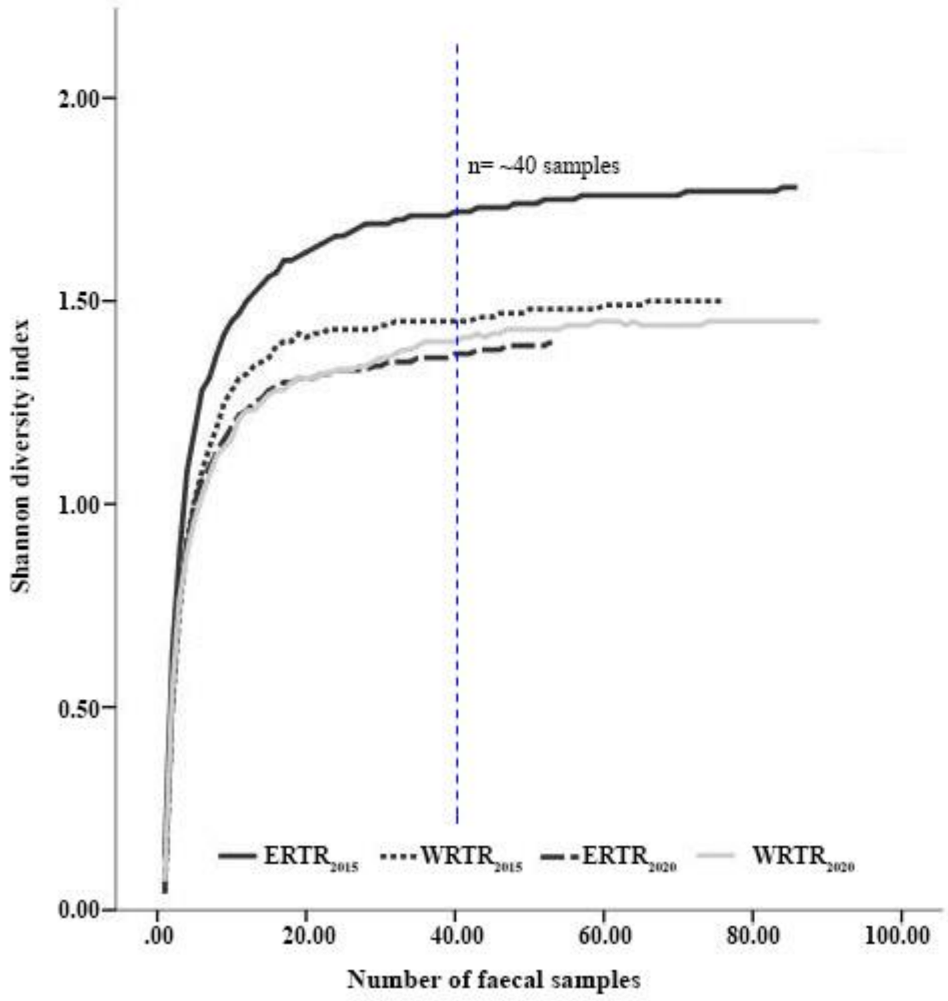

Supplementary Figure 3: Relationship of habitat NDVI values with leopard fGCM and fT3M levels. Scatter-plots showing (a) the positive association between NDVI values and fGCM levels and b) no significant association between NDVI values and fT3M levels.

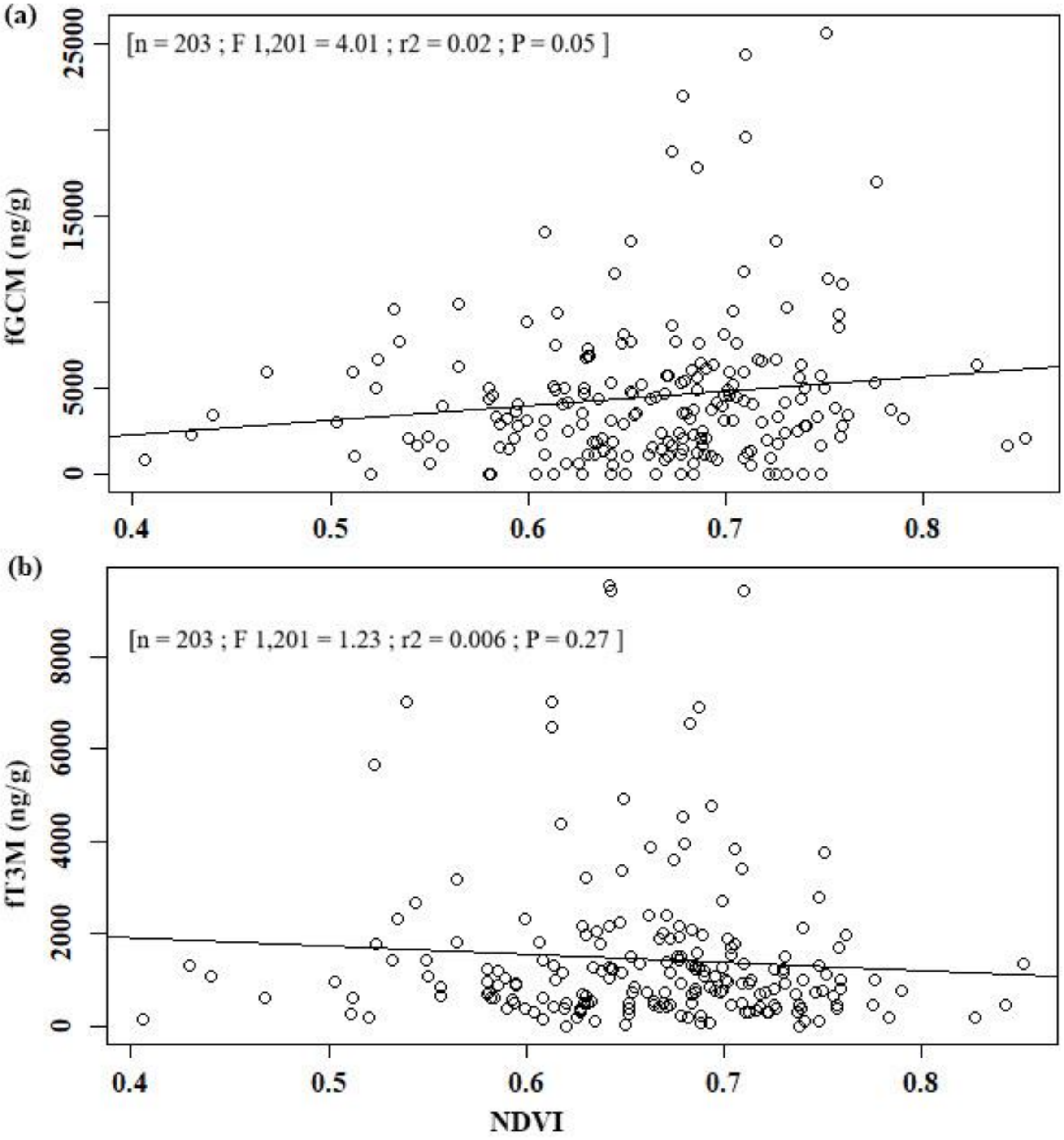

Supplement: Web_Material_coad039 [file web_material_coad039.pdf]
